# Effect of Standardized Fractions and Tiliroside from Leaves of *Tilia americana* on Depression Tests in Mice

**DOI:** 10.22037/ijpr.2019.1100883

**Published:** 2019

**Authors:** Yadid Chávez-Morales, Enrique Jiménez-Ferrer, Gabriela Belen Martínez-Hernández, Jaime Tortoriello, Rubén Román-Ramos, Alejandro Zamilpa, Maribel Herrera-Ruiz

**Affiliations:** a *Centro de Investigación Biomédica del Sur, Instituto Mexicano del Seguro Social (IMSS), Argentina 1, 62790 Xochitepec, Morelos, México. *; b *Doctorado en Ciencias Biológicas y de la Salud, División de Ciencias Biológicas y de la Salud, Universidad *; c *Autónoma Metropolitana (UAM), Iztapalapa, San Rafael Atlixco No.186, Col. Vicentina 09340, Iztapalapa, México D.F., México.*; d *Departamento de Ciencias de la Salud, División de Ciencias Biológicas y de la Salud, *; e *Universidad Autónoma Metropolitana- Iztapalapa, San Rafael Atlixco No.186, Col. Vicentina 09340, Iztapalapa, México D.F., México.*

**Keywords:** Tilia Americana, Flavonoids, Tiliroside, Antidepressant, FST, TST

## Abstract

Depression affects more than 300 million people worldwide, represents one of the leading causes of disability worldwide. Depression treatment is based on the use of tricyclic antidepressants, selective serotonin reuptake inhibitors. These drugs, although clinically effective, have also been shown to have delayed onset activity and produce significant adverse side effects. Medicinal plants are presented as a source of study in the search for therapies. This study was aimed to assess the antidepressant effect (on forced swimming test -FST- and tail suspension test -TST-) of different fractions and tiliroside from *Tilia americana*. The organic fractions (FAC1-1, FAC1-2) and aqueous fractions (FAqC2-1, FAqC2-3) were obtained by column chromatography and the HPLC analysis allowed the standardization based on the concentration (mg/g) of several compounds: FAqC2-1 with tiliroside 20, quercitrin 41.7, and quercetin glucoside 73.8; FAqC2-3 with tiliroside 2.4, quercitrin 16.6 and 7-O-luteolin glucoside 35.9; FAC1-1 caffeic acid was quantified with 7.87 ; FAC1-2 with tiliroside 24.7 and quercitrin 19.8. Each fraction was tested in ICR mice at different dose in the FST and TST, as well as in the open field test (OFT); tiliroside was isolated and tested in such assays (at 0.05, 0.1, 0.5, and 1.0 mg/kg). All fractions were active, the better was FAC1-2, and induced a dose-dependent effect on FST with an ED_50_= 2.59 mg/kg and Emax = 175.4 sec; with a sedative effect in OFT. Tiliroside with like-antidepressant activity, showed a dose-response behavior (ED_50_= 0.04 mg/kg and Emax = 121.42 sec for FST; ED_50_= 0.014 mg/kg and Emax = 78.28 sec for TST).

## Introduction

Depression can be considered as a syndrome that involves episodes of sadness, loss of interest, pessimism, negative beliefs about oneself, lack of motivation, passive behavior, sleep disorders, and suicidal thoughts, among others. It has become a serious health problem, especially when it is of long duration and with moderate or severe intensity. It also, causes the affected person to suffer greatly, substantially impairing the ability to function at work, at school, and in the family (1). According to the World Health Organization (WHO), depression is a common mental disorder that affects more than 300 million people worldwide. Approximately, 800,000 people die because of suicide each year, and this problem is the second leading cause of death in young people (2). It is also reported that it will be the second leading cause of disability in 2020 (1, 3), and therefore, has become a high priority on the global public health agenda (4). WHO indicates that prevention and appropriate treatment of depression may reduce suicide rates (5).

Although there are treatments for this condition, the effectiveness of common antidepressants is relatively low, as it can take weeks, months or even years to observe the desired effect. Thus, only approximately 47% of the patients respond to therapy, and only 33% reach remission with first-line therapy, including selective serotonin reuptake inhibitors (SSRIs). The great majority of patients recovered from a depressive episode will experience recurrence (6). 

In addition, SSRIs, monoamine oxidase inhibitors (MAOIs) and tricyclic antidepressants (TCA), cause major health problems such as hepatitis, blurred vision, dry mouth, increased blood pressure, hallucinations, loss of appetite, sexual dysfunction, among others (7).

Consequently, there is a continuing search for adequate and effective treatments for depression, which may have greater benefits than existing medications. In this sense, medicinal plants represent an important topic of the study (7-8). Therefore, traditional medicine throughout the world plays an essential role in health care, as it is used as a basis or complement in the treatment of various diseases. In fact, WHO estimates that three-quarters of the world′s population rely on medicinal plants to reduce their medical problems (1). 

In Mexico, *Tilia americana* var. *mexicana* (Schltdl.) Hardin is popularly known as *cirimbo*, *sirimo*, *tila*, *tilia*, *tirimo*, *tzirimo*, and *tzirimu*, depending on the region (9). It is a tree with a straight stem and smooth bark, of 18-35 m of height, with a period of flowering from April to June. This plant is widely used throughout the country, in various ways, such as infusions of the leaves with or without inflorescences to treat nervous disorders. It has been reported that the fresh plant is ″more active to sleep″, ″to calm nervous feelings or tension and restore sleep″, and to treat a headache (9-11). In addition,* T. americana *is effective in the treatment of ″nervousness″, which refers to a popular disease recognized by traditional Mexican medicine, which presents symptoms of restlessness, weakness, appetite disorders and mood*.* All of them are associated with the symptomatology of depression in Western medicine (11-13). 

The sedative and anxiolytic effects of this plant have been demonstrated in previous studies, in which different extracts were administered to mice exposed to various behavioral tests such as the elevated plus maze (EPM) and OFT, among others (14-17). In addition, the organic and aqueous extracts of the inflorescences showed anticonvulsive activity (18) and also neuroprotective effect in mice with ischemic brain damage (19). A standardized fraction in its flavonoid content, called F1C (16), has an anxiolytic effect in EPM by interacting, at least in part, with the drugs that modulate the serotonergic system (20). 

Different saccharides including fucogalactoxyloglucan, L-fucose, D-galactose, D-xylose, and D-glucose have been identified in leaves of *T. americana*. Some terpenes such as βsitosterol, and flavonoids such as quercetin 3-O-glucoside, and quercetin 1,3,7-O-diglucoside have also been identified (21). The flavonoids tiliroside, rutin, quercitrin, kaempferol, and quercetin and its glucosides (quercetin 3-O-glucoside, and quercetin 1, 3-Oglucoside) were isolated from the bracts of this species (16-17). 

In the present work is showing the antidepressant and sedative effects of fractions and tiliroside from leaves of *T. americana* using the FST, TST, and OFT tests*. *Each fraction was standardized by HPLC based on its contents of tiliroside (Til), quercetin rhamnoside (quercitrin), quercetin glucoside and 7-O-luteolin glucoside. Caffeic acid was identified in one of these fractions.

## Experimental

*Reactives and drugs*

Tween 20, imipramine (IMI, ≥99% -TLC), and acetonitrile were purchased from Sigma-Aldrich Chemical Co. (St. Louis, MO, USA). Methanol and ethyl acetate (EtOAc) were purchased from Mallinckrodt Baker, Inc. (Phillipsburg, NY, USA). The crude extract, as well as fractions, were separated and analyzed by column chromatography and thin-layer chromatography (TLC). Silica gel (70–230 mesh), reverse-phase silica gel C18 (40–63 μm, Merck), and silica gel on plates (CP) were obtained from Merck KGaA. The developers used were 2-aminoethyl diphenylborinate (detection of flavonoids) and 4-hydroxy-benzaldehyde (detection of terpenes), were obtained from Sigma-Aldrich. 

*Plant Material*

Only leaves without inflorescences of *T. americana* were collected in Mexicapa, Ocuilan, State of Mexico, Mexico, in July and August 2013. They were dried and ground twice in a Pulvex electric mill. Afterwards, 2.0 kg of material were weighed and placed in a 20 L transparent glass container for maceration with solvents. The plant material was taxonomically identified in the Herbarium of the Instituto Mexicano del Seguro Social-IMSS, located at the Centro Medico Nacional Siglo XXI. It was identified as *Tilia americana *var. *mexicana *with a voucher number 15099. To obtain extracts and a fraction rich in flavonoids, the methodology proposed by Herrera-Ruiz was carried out (16, 20). 

*Preparation of Extracts*

The dried, milled material was initially macerated with 5 L of *n*-hexane. The plant material recovered was then extracted with the same volume of methanol (24h × 2). Each extract was evaporated to dryness with a rotary evaporator (Laborota 4000, Heidolph, Germany). 

The methanol extract (150 g) was subjected to a bipartition separation method with ethyl acetate (AcOEt) and water (H_2_O), which resulted in two phases: organic (FA; of ethyl acetate), and aqueous, (FAq). The ethyl acetate fraction (30 g, FA) was purified by open-column chromatography (OCC) using 3.0 cm diameter, 60 cm length columns previously packed with silica gel (0.063-0.2 mm, 300 g). The elution system consisted of a gradient of CH_2_Cl_2_/CH_3_OH. Thin-layer chromatography (TLC) was used for separation. Samples with similar chemical compounds were grouped generating FaC1-1 (630.9 mg; 2.1%); FaC1-2 (228.3 mg; 0.76%); FaC1-3 (331.9 mg; 1.1%); and FaC1-4 (90.8 mg; 0.3%) fractions. Thus, it was possible to combine chemically similar products that were grouped into four fractions from low to high polarity: FAC1-1, FAC1-2, FAC1-3 and FAC1-4. 

Separation of polar compounds present in the aqueous fraction was performed by OCC packed with reverse phase silica (Merck). In this case, a H_2_O-acetonitrile gradient system was used as the mobile phase. The extracts were concentrated by the reduced pressure followed by lyophilization (freeze drying). Three fractions were obtained from this separation: FAqC2-1 (6500 mg, 21.7 %), FAqC2-2, (200 mg; 0.66%), and FAqC2-3 (100 mg; 0.33%). All of them were stored at -4 °C until their biological evaluation or chemical separation. The FAC1-2 fraction was selected for chemical analysis using reverse-phase open column (1.0 cm diameter, 30 cm length; 15g). The elution system started with H_2_O, and the polarity of the solvent was gradually modified with methanol (MeOH). The fractions were monitored by HPLC, which allowed the detection of different compounds by using standard substances. Each collection was monitored by TLC, and samples with similar chemical compounds were grouped. Ceric ammonium sulfate was used to develop the plates, while the specific compound 2-aminoethyl diphenylborinate (1% methanol) was used to reveal the presence of flavonoids, with coloring from yellow to bright orange (22). 

*HPLC analysis*

The chromatographic analysis was developed using a HPLC system equipped with a 2695 Separation Module, a Waters 996 photodiode array detector, and Empower Pro software (Waters Corporation, USA). The compounds were separated on a LiChrospher 100 RP-18 column (4 mm × 250 mm i.d., 5-µm particle size) (Merck, Darmstadt, Germany). The mobile phase consisted of a 0.5% aqueous solution of trifluoroacetic acid (solvent A) and acetonitrile (solvent B). The gradient system was as follows: 0-1 min, 10% B; 1-2 min, 15% B; 3-5 min, 20% B; 5-10 min, 70% B; 10-13 min, 60; 14-20 min, 100% B; 21-23 0% B; 24 min, 0% B. The flow rate was maintained at 1 mL min^−1^ and the injection volume was 10 µL. Absorbance was measured at 350 nm. 

The compounds were identified by values in their UV spectra: flavonols (λ= 210, 250, and 350 nm), tiliroside (λ = 260 and 314 nm), and polyphenol (λ = 325 nm). The previously isolated compounds (tiliroside and quercitrin) as well as quercetin3-glucoside, caffeic acid, and 7-O-luteolin glucoside (Sigma-Aldrich, St Louis MO, Purity: > 98%; >98% and an analytical standard, respectively) were used as reference standards and its calibratioin curve was built using four ascendant concentrations (6.25, 12.5, 25 and 50 µg/mL).

*Isolation of Tiliroside (Til)*

Based on the chemical analysis by HPLC, from 12.7 g of the fraction FAC1-2 the flavonoid tiliroside was isolated by using different chromatographic columns: normal phase (silica gel 0.063-0.2 mm, 120 g, Merck, Darmstadt, Germany) and using an elution system dichloromethane: methanol, collecting 18 fractions. The fractions were monitored by TLC. Fraction number 8 was selected due to the quantity of tiliroside. Then it was subjected to separation by column chromatography using dichloromethane: methanol, obtaining 19 fractions. Fraction 10 was selected and separated in a chromatographic column reverse phase (100 RP-18, purchase in Merck, Darmstadt, Germany). In this process, the mobile phase was water: acetonitrile. Of the total of 68 fractions from this column, on the last 14, a yellow amorphous product, that was identified as tiliroside (0.18 g). 

*Behavioural Activity*

The experimental protocol was approved by the IMSS local Health Research Committee (registry number R-20131701-69). All procedures were conducted in accordance with the Official Mexican Norm (NOM-062ZOO-1999) regarding technical specifications for production, care, and use of laboratory animals, and the Guide for the Care and Use of Laboratory Animals. For the euthanasia of the mice, the overdose technique with barbiturates (sodium pentobarbital) was selected, which is accepted by the official Mexican Norm with the number NOM-062-ZOO-1999 (Technical specifications for the production, care and use of animals from laboratory). 

*Experimental Animals*

Male ICR mice weighing 30-35 g were used for the study and randomly assigned to the different experimental groups and were obtained from Envigo México (Envigo RMS S.A.). They were allowed to adapt to the laboratory environment for at least two weeks prior to experiment initiation. |The animals were housed at 25 °C under a 12-hour light/12-hour dark cycle, with free access to water and standard food. The tests were conducted in a special noise-free laboratory at 25 °C with red light, and the number of individuals per group was six, and each mouse was used for only one test. All experimental procedures were carried out from 8:00 a.m. to 1:00 p.m.

*Treatments and study design*

Imipramine (IMI, 15 mg/kg) was used as the standard antidepressant drug (positive control group), and Tween 20 solution (TW, 1%, Sigma) was employed to treat negative control groups. Treatments from *T. americana* were: ethyl acetate extract (AcOEt) at a dose of 25 mg/kg, and fractions obtained from FAC1: FAC1-1, FAC1-2, FAC1-3, and FAC1-4; and those from FAqC2: FAqC2-1, FAqC2-2, and FAqC2-3.

*Experimental procedures*

Initially, the AcOEt extract, the fractions FAC1, and FAqC2 from the chromatographic columns were tested. A dose of 25 mg/kg of each was orally administered to the mice which were exposed to the FST. Due to the results obtained, different doses of the most active fraction (6.25, 12.5, 25, 50, and 75 mg/kg) were then evaluated in the FST, TST, and OFT. The flavonoid isolated, Tiliroside (Til) was also evaluated on the same behavioral tests to 0.05, 0.1, 0.5, and 1.0 mg/kg. 

*Forced Swimming Test (FST)*

The apparatus consisted of a clear Plexiglas cylinder (20 cm high × 12 cm diameter) filled with water (24 ± 1 °C) to a depth of 15 cm. In the pre-test session, each animal was placed individually into the cylinder for 15 min, 24 h prior to the 5 min swimming test. Treatments from leaves of *T. americana*, imipramine (IMI), and the vehicle were administered three times: immediately after the initial 15-min pre-test, 18, and 1 h prior to the swimming test. During the test session a trained observer registered the immobility time, considered when the mouse made no further attempts to escape, apart from the movements necessary to keep its head above the water. It was suggested that immobility reflected a state of lowered mood in which the animals had given up hope of finding an exit and had resigned themselves to the experimental situation (modified from 23).

*Tail Suspension Test (TST)*

The mice were suspended individually by their tail from a wood rod fixed 50 cm above the surface of a table. The tail was fixed with adhesive tape for 6 min, and during this period was recording the immobility time (when the animal does not make attempts to escape) (24).

*Open Field Test (OFT)*

The open-field area was made of acrylic (transparent walls and black floor, 30 × 30 × 15 cm) divided into nine squares of equal area. The open field was used to evaluate the exploratory activity of the animal. The observed parameters were the number of squares crossed with the four paws (total crossing, TC) and the number of rearing (R) (25, 26). 

*Statistical Analysis*

For statistical analysis of the results, an Analysis of variance (ANOVA) followed by a Dunnett post-test was utilized and a *p* < 0.05 for defining significant differences among the groups was considered. SPSS version 11.0 statistical software was used for such analysis.

## Results

*Preparation and chromatographic fractionation of methanolic extract*

Leaves of *T. americana* (1 Kg) were extracted by maceration in methanol, yielding 169.1 g (8.45%) of extract. 150 g of this extract were separated by bipartition with ethyl acetate / water. Purification of the ethyl acetate fraction (TaAcOEt) yielded four sub-fractions (FAC1-1, 2.13%, FAC1-2, 0.76%, FAC1-3, 1.13%, FAC1-4, 0.31%), while the chromatographic separation of the aqueous fraction gave rise to three sub-fractions (FAqC2-1, 21.72%, FAqC2-2, 0.66% and FAqC2-3, 0.33%).

*Identification and standardization of compounds by HPLC*

**Figure 1.**
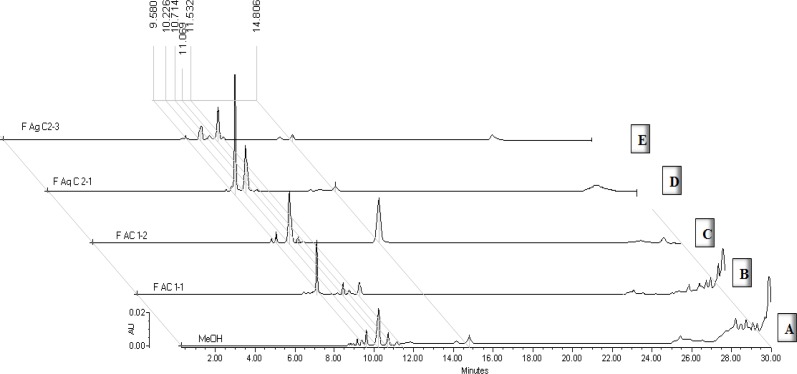
Chromatographic profile by HPLC of several fractions from leaves of *Tilia americana *(wave length λ= 350 *n*m for flavonoids and λ= 325 *n*m for caffeic acid). Tiliroside, RT=14 min; Quercitrin, RT= 10 min; 3-O-Quercetin glucoside, RT = 9.5 min; 7-O-Luteolin glucoside, RT= 11.06 min and caffeic acid, RT = 9.3 min

Crude extract was analyzed by HPLC (Figure 1A), main flavonoid compounds were detected, and the most significant peaks at 10.2 min and 14.6 min corresponded to quercitrin and tiliroside, respectively. These compounds had already been described by our research group (16) (20). The concentration of some flavonoids was determined by chromatographic analysis of each fractions.

Liquid-liquid extraction of the crude extract allowed separating the polar compounds present in the aqueous fraction (FAq) from the low-polarity compounds of the ethyl acetate fraction (FA). 

The fractions resulting from the chromatographic purification of FA were analyzed chemically and pharmacologically and showed a difference in their chemical composition. For example, FAC1-1 fraction (Figure 1B) contained mainly a peak at 9.3 min, which showed the same retention time and the same UV spectrum (λ = 210, 325 nm) than caffeic acid. Fraction FAC1-2, on the other hand, contained mainly quercitrin (Figure 1C, RT = 10.1 min, λmax = 210, 255, 350 nm) and tiliroside B (RT = 15.5 min, λmax 210, 266, 314 nm) in a ratio of 1: 1.

Moreover, from the fractions obtained of the aqueous purification, it was observed that the more polar fraction (FAq-C2F1, Figure 1D) consisted mainly of two peaks at 9.5 min and 10.1 min, which showed a retention time and UV spectra λmax = 212, 255, 355 nm and 212, 255, and 350 nm characteristic of quercetin-3-glucoside and quercitrin, respectively. Fraction FAq-C2F3 showed a 10.1 min peak of quercitrin and a major peak at 11.06 min with the same RT and UV spectra as 7-O-luteolin glucoside (Figure 1E).

The quantitative chemical analysis of the extract and the pharmacologically evaluated fractions, allowed to establish the content of these flavonoids in each treatment (Table 1). The concentration of tiliroside in FAC1-2 was 24.7 mg/g, in the organic fraction of 4.8 mg / g and in the complete extract of 0.98 mg/g. While the concentration of quercitrin was 19.8 mg/g in FAC1-2; of 16.6 mg/g in the organic fraction and 6.2 mg / g in the methanol extract. 

The content of these compounds in FAqC2-1 and FAqC2-3 were as follows: 20.0 and 2.4 mg/g of tiliroside and 41.7 and 16.6 mg/g of quercitrin, respectively. Other compounds were quantified for each of these fractions, such as quercetin glucoside in FAqC2-1 with 73.8 mg/g, and 7-O-luteolin glucoside in FAqC2-3 with 35.96 mg/g. 

Finally, the major peak in FAC1-1 corresponding to caffeic acid and was quantified in such sample (Table 1).

*Isolation of tiliroside (TIL)*

Fraction FAC1-2 (0.98 g) was subjected to reverse phase open column chromatography. This fraction was placed in a glass column (400 x 20 mm) pre-packed with the stationary phase (RP-18 Lichroprep, 40-63 μm, 10 g) and eluted with a mixture water / acetonitrile. The samples of 10 mL were collected, pooled based on their chemical similarities by thin layer chromatography and HPLC analysis. All fractions containing a chromatographic profile similar to the tiliroside standard were pooled, from them the tiliroside (Rt= 14 min, λ= 210,266, 314 nm) compound was isolated. The identity of was made by direct comparison of retention time, UV analysis and ^1^H NMR data with those previously reported for this polyphenolic compound (16).

*Evaluation of different Fractions of T. americana in depression tests*

*Forced Swimming Test (FST)*

Table 2 shows the results obtained after administration of Tween 20 solution (TW, 1%, Merck) used as vehicle (VEH), this treatment caused an immobility time of 219 ± 20.6 sec in the FST. This effect was counteracted at the dose of 15 mg/kg of the antidepressant IMI and showed a significant difference (*p* < 0.05) with respect to the VEH group. 

The animals administered with a dose of 25 mg/kg of AcOEt extract showed immobility time similar to IMI, but significantly different from the animals of the VEH group (*p *< 0.05). It was observed that only FAC1-1 and FAC1-2 fractions at a dose of 25 mg/kg induced a significant reduction in the immobility time of the mice in the cylinder of water compared with the VEH group (*p* < 0.05, 

Table 2). 

FAC1-3 and FAC1-4 fractions did not cause significant changes in the immobility parameter with respect to the control group (*p *> 0.05). FAqC2-1 and FAqC23 fractions to 25 mg/kg, significantly reduced the immobility time of mice in the FST, compared to those in the VEH group (Table 2, *p* <0.05). The FAqC2-2 did not induce changes compared with the control group (*p* >0.05).

*Tail Suspension Test (TST)*

Considering the results obtained in the FST, the active fractions were evaluated at the same dose in TST. The animals administered with the vehicle showed an increase in immobility time, while those receiving IMI at 15 mg/kg, decreased this parameter, both groups were statistically different (*p* < 0.05, Table 2). All fractions evaluated in this test, showed antidepressant effects similar to IMI, and were significantly different from the control group (Table 2; *p* < 0.05).

*The Open Field Test (OFT)*

The active fractions in FST and TST tests were evaluated in the OFT test. Data from this study showed that FAC1-1, FAC1-2, and FAqC1-1 fractions at 25 mg/kg significantly decreased TC and R parameters, in comparison with the VEH group (*p* < 0.05). FAqC2-3 fraction at the same dose, and IMI at a dosage of 15 mg/kg, did not modify the behavior of the mice in this assay (Table 3).

*Effect of different dose of the fractions in FST*

**Figure 2:**
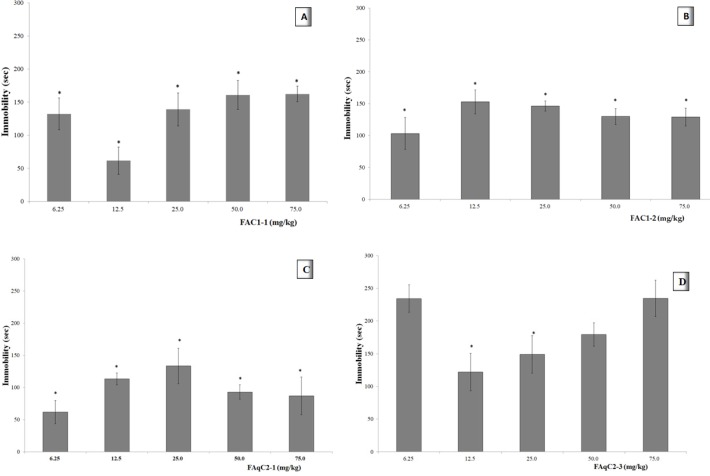
Dose-response curve of organic fractions: FAC1-1 (**A**) and FAC1-2 (**B**); aqueous fractions: FAqC2-1 (**C**) and FAqC2-3 (**D**) from leaves of *Tilia americana*, on immobility time of mice in FST**. Insert in A: **Imipramine (IMI 15.0 mg/kg), VEH (Tween 20 solution 1%). ANOVA, with post-test Dunnet with *p <0.05 (n = 6, *ẋ *±SD), in comparison with the VEH

Figure 2A shows that all doses of the FAC1-1 induced reduction in the immobility time of the mice in the cylinder. This effect was significantly different from the control group (VEH, *p* <0.05; see insert) at each dose. The immobility time, was less at a dose of 12.5 mg/kg. When doses were higher, a response similar to the dose of 6.25 mg/kg was observed.

The animals administered with a dose of 6.25 mg/kg of FAC1-2 (Figure 2B), showed reduction in the immobility time in the cylinder with water. When the dose was increased, a lower effect was observed. The data obtained in all groups indicated a statistical difference compared with the control group (*p* <0.05). For the range of 12.5 to 75 mg/kg the Emax=111.3 sec and ED_50_= 0.83 mg/kg. 

The dose of 6.25 mg/kg of FAqC2-1 caused a reduction in the immobility time in the FST, nevertheless, when the dose was increased up to 25 mg/kg, a lower effect was observed. All groups were different from the VEH group (Figure 2C, *p* <0.05). 

Dose of 6.25 mg/kg and 75 mg/kg of FAqC2-3 did not induce changes in comparison with the VEH group (*p* > 0.05). Intermediate doses showed higher immobility times than the other fractions at the same doses. 

Furthermore, the effect decreased as the dose increased, although these three doses (12.5, 25, and 50 mg/kg) were statistically different compared with the vehicle group (Figure 2D, *p* < 0.05).

*Effect of different dose of the fractions in TST*

**Figure 3:**
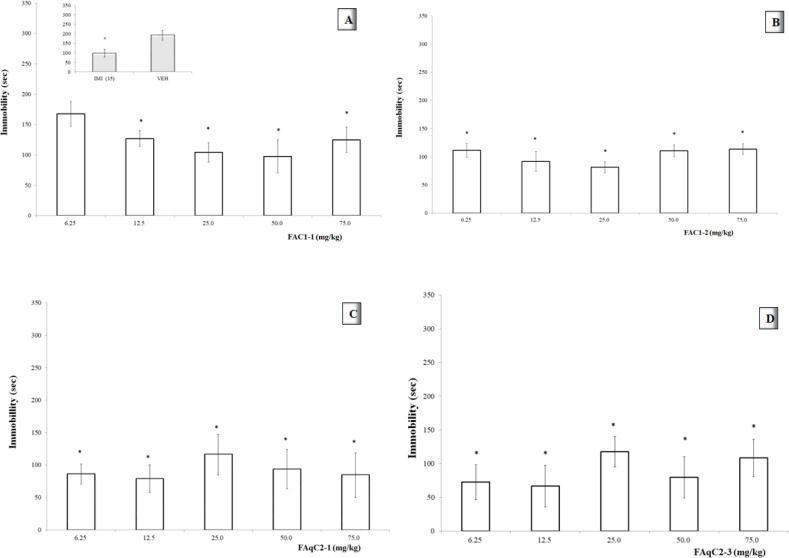
Dose-response curve of organic fractions: FAC1-1 (**A**) and FAC1-2 (**B**); aqueous fractions: FAqC2-1 (**C**) and FAqC2-3 (**D**) from *Tilia americana *leaves, on parameter of immobility of mice in TST**. Insert in A: **Imipramine (IMI 15.0 mg/kg), VEH (Tween 20 solution 1%). ANOVA, with post-test Dunnet with *p <0.05 (n = 6, *ẋ *±SD), in comparison with the VEH

The immobility time of the tail-suspended mice treated with a dose of 6.25 mg/kg of the FAC1-1 fraction was not significantly different from the VEH group (*p >0.05). However, higher doses induced a significant reduction (*p <0.05, Figure 3A) in this parameter, and a dose-dependent effect was observed in the center of the curve (12.5, 25, and 50 mg/kg), with an ED_50_= 2.53 mg/kg and an Emax= 82.22 sec. 

The FAC1-2 fraction caused a significant decrease in the immobility parameter at all doses compared with the VEH group (*p* <0.05, Figure 3B). However, a dose-response tendency was detected in the range of lower doses (6.25 to 25 mg/kg), which resulted in an ED_50_= 1.38 mg/kg and an Emax = 65.88 sec. 

As the other fractions, FAqC2-1 caused a significant reduction in the immobility activity of the mice compared with the control group (*p* <0.05). Figure 3C shows that activity at the median dose (25 mg/kg) was slightly lower than activity at lower doses. However, there was a tendency to increase the effect at higher doses. An ED_50_=1.14 mg/kg and Emax= 74.29 sec, were calculated in the range of higher doses. 

In the case of the FAqC3 fraction, since there was not a defined pattern at higher doses, two lower doses were also evaluated (1.56 mg/kg and 3.125 mg/kg) to determine if there was a dose response tendency in this range. 

Figure 3D shows that the effects of the FAqC3 fraction on the immobility parameter were persistent at lower doses. In addition, as the dose was increased up to 12.5 mg/kg, a dose-dependent effect was observed, resulting in an ED_50_= 0.18 mg/kg and Emax= 66.74 sec.

*Effect of different dose of the fractions in TST*

The administration of IMI to 15 mg/kg did not modify the TC or R parameters compared with the VEH group (*p* >0.05). All doses of FAC1-1 and FAC1-2 organic fractions caused a decrease in both TC and R parameters, with a significantly different change compared with control group (Table 4, *p* <0.05). 

FAqC2-1 and FAqC2-3 fractions produced a decrease in TC and R parameters only at some doses. Thus, there was not a defined pattern of the spontaneous motor activity (Table 4). 

*Effect of Til on FST, TST and OFT*

**Figure 4:**
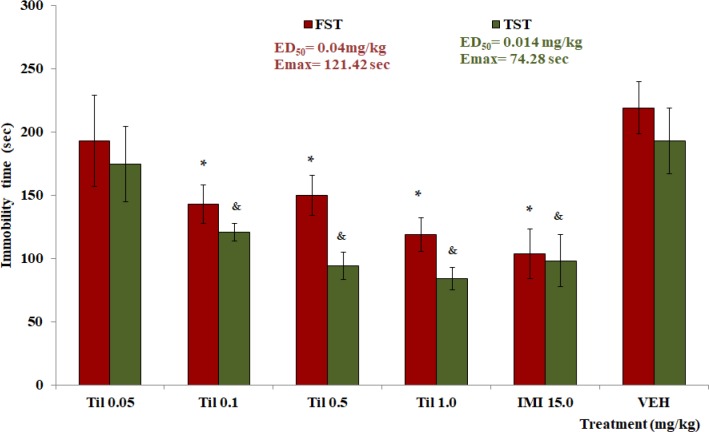
Different doses of tiliroside (Til) on parameter of immobility time of mice on FST and TST . Imipramine (IMI 15.0 mg/kg), VEH (Tween 20 solution 1%). ANOVA, with post-test Dunnet with *p <0.05 (n = 6, *x *±SD) in comparison with VEH of FST and &*p *<0.05 (n = 6, *x *±SD) in comparison with VEH of TST

Doses of 0.1, 0.5, and 1.0 mg/kg of Til, showed a decrement of the immobility time on FST and TST tests. The data were significantly different with the VEH group in each test (*p <0.05 comparated with VEH in FST; ^&^*p* <0.05 comparated with VEH in TST). The ED_50_ and Emax were calculated for tiliroside (Figure 4). All doses of tiliroside induced a significant decrease in TC and R in the OFT, respecting the control group (*p <0.05, Table 5).

## Discussion

*T. americana *is used in Mexico for the treatment of CNS-related disorders such as insomnia, headache, and people use it for ″nerves″, a term that includes disorders of mood, sleep, eating and weakness, among others, that may be associated with depression. The present work shows that different fractions of leaves of this plant have a chemical profile like that of bracts and flowers (16, 17, 19), and that those possesses antidepressant and sedative effect. 

The FST is perhaps the most used assay due to its relative reliability across laboratories, and it is based on the observation that when the mice are placed in a cylinder with water they initiate escape-oriented movements, and eventually assume a characteristic immobile posture or with minimal movements. Such behavior is modified by the administration of antidepressants, so that the subjects persist in performing active movements that lead them to try to escape (23, 27). Furthermore, the test has a predictive capacity, which allows it to detect antidepressant effect of a wide variety of drugs (28). TST shares its experimental basis with the FST and provides useful information on the individual′s ability to cope with acute stress situations, which can be modified by pharmacological or even genetic manipulation (29). 

Fractions FAC1-2, FAqC2-1, and FAqC2-3 from *T. americana *were able to induce antidepressant activities on both pharmacological tests, and it is caused by the flavonoid content. Nevertheless, FAC1-2 is the only one that induced a dose-dependent effect in the FST. 

With respect to the aqueous fractions, FAqC2-1 induced an important effect in FST, however, at high doses this effect decreased. This fact could be associated with the proportion of different flavonoids, for example in this fraction the compound most abundant is quercetin glucoside, an in second place is quercitrin, whereas tiliroside was found in less concentration compared with both. 

The antidepressant effect of FAqC2-3 was even lower. Moreover, it was remarked that the higher administered dose, the lower the effect it was produced, and finally the dosage of 75 mg/kg did not cause any activity in the FST. This treatment was characterized by a low concentration of tiliroside and quercitrin, but with high concentration of 7-O-luteolin glucoside and absence of quercetin glucoside. 

It was not possible to detect flavonoids in the FAC1-1, but caffeic acid was identified as the major peak in FST, and this fraction does not induce a dose-dependent behavior, and in fact there is a marked biphasic behavior in which the best effect is observed at 12.5 mg/kg. One could consider that caffeic acid was responsible for the antidepressant activity in this case. 

The results observed in TST indicate that all fractions induced not only a biphasic effect, but also a dose-dependent effect in some dose ranges. In those fractions containing tiliroside and quercitrin, it was observed that the presence of tiliroside, quercitrin, and quercetin glucoside in the FAqC2-3 induced a level of efficacy similar to that of the FAC1-2 fraction, and a higher potency to fraction FAqC2-3 observed from the dose of 1.56 mg/kg. In this term, fraction FAC1-1 which contains caffeic acid, turned out to be slightly less active than the others, although it maintained a good antidepressant activity in the TST.

All treatments showed different pharmacological behavior in the assays FST and TST, which could be due to chemical composition, and probably to the characteristics of these tests. In addition, it is likely that the routes by which they carry out its effects are diverse. And when doses are modified, then the proportion of active components is also modified. For example, TST possesses higher sensibility to detect antidepressants that selectively inhibit serotonin reuptake (SSRIs) (29). Thus, regardless of the composition of the fractions of *T. americana*, the antidepressant effect was present and it is probable that this activity is the result of a modulation of different mechanisms, but it requires further research.

It has been demonstrated that antidepressant-response curves may be different between the TST and FST. This could be an explanation for the U-shaped dose-response curve behavior for the dose range considered. For instance, it is relatively usual that some compounds induce a biphasic response in the FST, while this arrangement is not so common in the TST analysis. An example is the IMI curve: of the dose range from 2.5 to 45 mg/kg administered to C57BI/6 mice, only the doses of 5, 10, and 15 mg/kg produced effects on the immobility parameter in the FST, while the lowest and highest doses did not produce any effects. In contrast, a linear response, typical of a classic curve (30), was observed in the TST for this drug. Treatments of *T. americana* caused a biphasic response in both tests. However, while the dose range evaluated of the four fractions showed a linear tendency in the TST, not all of them showed this behavior in the FST.

It is widely recognized in a diversity of biological models, that flavonoids represent secondary metabolites with a great variety of pharmacological effects, such as the antidepressant activity (31). As indicated, quercitrin constitute part of the composition of the *T. americana* antidepressant treatments. It was reported that quercitrin, for example, and kaempferol, both isolated from the plant *Opuntia ficus* *indica* var. *saboten* (Cactaceae), produced antidepressant effects in the TST and FST when a daily dose of 30 mg/kg was orally administered during 14 days to mice exposed to the stress restraint model (32). 

A methanol extract from the flowers of *Hypericum montbretti* Spach. (Guttiferae Clusiaceae) with a high concentration of quercitrin showed antidepressant effects in the TST and FST at doses of 100 and 250 mg/kg. Even though authors attributed the antidepressant effect to the high concentration of rutin (1519 ppm), quercitrin could have also been an important part of this effect since it possessed the second highest concentration (784 ppm) in the extract (33); and its antidepressant characteristic has also been demonstrated (32). In addition, another study demonstrated that the doses of quercitrin isolated from a flavonoid fraction of *Hypericum perforatum* at doses of 0.6 and 1.3 mg/kg, did not show antidepressant effects in the FST (34). This could probably have been due to the relatively low dose employed and the duration of administration compared with Park’s study (32). This information together indicates that quercitrin possesses antidepressant effects that may depend on the dose and duration of administration, and even on the pharmacological interaction established between the flavonoids of a fraction or extract. 

Luteolin is another flavonoid, isolated from *Cirsium japonicum* (Compositae). A dose of 10 mg/kg was orally administered to mice causing reduction in the immobility time in the FST and providing chloride ion (Cl^-^) flux through the GABA-A receptor-ionophore complex as a possible antidepressant mechanism of action (35). While 7-O-luteolin glucoside was identified in *T. americana*, it is possible that this derivative of luteolin also has the antidepressant activity, while, quercetin glucoside is part of the anxiolytic fraction isolated from *T. americana* (20) and could be part of the actives from this plant with antidepressant characteristics. 

Caffeic acid is found in diverse plants. Several studies using behavioral methods have demonstrated its antidepressant effects. For instance, the intraperitoneal administration of this compound (1.2 and 4 mg/kg) to male ICR mice caused a dose-dependent effect on the immobility parameter in the FST, with significant modification at a dose of 4 mg/kg. It was also reported that the probable mechanism of action is not related to monoamine re-uptake or to monoamine oxidase (MAO) inhibition (36). In the animals subjected to the FST, a significantly decrement on the expression of the mRNA for BDNF was observed, and this effect was counteracted with the administration of caffeic acid by intraperitoneal pathway (37), which established that this process is part of the mechanism of action by which this metabolite, provoke its antidepressant action. 

Locomotor activity was evaluated for all fractions from *T. americana* and doses in the OFT to demonstrate that reduction in the immobility time in the FST and TST was not a secondary consequence of a not specified locomotor stimulating action. Altogether, the results showed that none of the fractions increased the spontaneous motor activity of the mice in this test. 

This suggested that the effect of the increased mobility of the mice in the cylinder was not due to a motor increase. However, all doses of the FAC1-1 and FAC1-2 fractions induced a sedative effect since TC and R parameters were significantly lower than those of the control group. Hitherto, the studies about biological effects of *T. americana* reported its sedative effect evaluated in the OFT, or its boosting effect caused by pentobarbital (17). The data mentioned here confirmed this activity, but only for some fractions. To some extent, this may be related to the presence of significant concentrations of tiliroside in FAC1-2 and caffeic acid in FAC1-1 fractions. In the literature, the anxiolytic and sedative effect of a fraction rich in flavonoids from *T. americana* and its pharmacological interaction with serotoninergic neurotransmission system, mainly through with 5-HT1A, has been reported. It is necessary notice this action, due in that anxiolytic fraction the tiliroside is present as a principal compound. This is relevant to the extent that serotonin is involved in the pathological mechanism of depression, and all fractions of *T. americana* analyzed in this work were antidepressant in both tests. It is known that serotonin possesses more susceptibility not only to the antidepressant effect of SSRI drugs, but also to the sedative effect of agonists at 5-HT1A receptors (20). 

Tiliroside (kaempferol-3-β-(6″-*p*-coumaroyl)-βD-glucoside) isolated from *T. americana *was tested at different doses and was shown that this flavonoid possesses antidepressant activity in both behavioral assays, of a dependent-dose form, and it also induced a sedative behavior on the OFT, which is congruent with previous reports indicating the sedative capacity of the plant (14, 15). There are no data about the CNS activity of this compound. Only two studies, until now, indicated that tiliroside may be one of the active compounds that provide *T. americana* with the anxiolytic activity (16) (20), but this is not shown yet. In the previous paragraphs it was mentioned that kaempferol possesses antidepressant effects (32). This idea has been adopted in this study since tiliroside is a derivative of kaempferol. Therefore, it is probable that part of the antidepressant activity observed in fractions containing tiliroside is due to the presence of kaempferol (39). 

Moreover, there is evidence about that flavonoids and other polyphenols could be acting as pro-drugs. Referent this, it was showed that quercetin and kaempferol (range of dose of 0.1 to 2.0 mg/kg) have not anxiolytic activity when were administered by intraperitoneal pathway in the mice exposed to elevated plus maze test. 

Then the authors concluded that these flavonoids are pro-drugs (40). Also, several metabolites from degradation of these compounds such as, phloroglucinol and the hydroxyphenyl acetic acids (para- and meta-HPAA) and DOPAC (3,4 Dihydroxyphenyl acetic acid) have been described. And for caffeic acid, one of the derivatives of its intestinal catabolism is the *m*-hydroxyphenylproprionic acid. In accordance with this proposal, it must be taken into account that the bioavailability and then the biological activity related to it after oral administration are influencing on the gastrointestinal tract, due to flavonoid structure, interactions with the food matrix, the activity of hydrolytic enzymes, the composition of the microbiota, and intestinal epithelial cell transporters (41). 

**Table 1:** Standardization data of treatments from *Tilia americana *var. *mexicana*

**Products of ** ***T. americana***	**Tiliroside (mg/g)**	**Quercitrin (mg/g)**	**Quercetin glucoside (mg/g)**	**7-O-Luteolin glucoside (mg/g)**	**Caffeic acid (mg/g)**
FAC1-1	ND	ND	ND	ND	7.87
FAC1-2	24.7	19.8	ND	ND	ND
FAqC2-1	20.0	41.7	73.8	ND	ND
FAqC2-3	2.4	16.6	ND	35.96	ND

ND= no detected.

**Table 2:** Effect of fractions from leaves of *Tilia americana *on the immobility time of mice on FST and TST

**Treatment**	**Immobility time (sec)**	
25 mg/kg	FST	TST
TaAcOet	101.8 ±16.2	----
**Organic fractions**		
FAC1-1	139.0 ±24.75*	104.1 ±16*
FAC1-2	145.0 ± 7.8*	81.5 ±9.2*
FAC1-3	197.8 ±29.21	----
FAC1-4	204.6 ±22.45	----
**Aqueous fractions**		
FAqC2-1	126.6 ±19*	114.6 ±33*
FAqC2-2	219.6 ±21	----
FAqC2-3	149.0 ±28.7*	117.6 ±22*
**Controls**		
IMI (15 mg/kg)	103.5 ±19.34*	98.0 ±20*
VEH (100 µL/10 g)	224.7 ±15.6	192.8 ±25

**Table 3:** Effect of antidepressant fractions from leaves of *Tilia americana*, on total crossings (TC) and rearings (R) of mice in OFT

**Treatment (25 mg/kg)**	**TC**	**R**
FAC1-1 (25)	97.6 ±13.1*	32.7 ±6.4*
FAC1-2 (25)	102.6 ±7.5*	45.0±11.4*
FAqC2-1 (25)	120.8 ±9.6*	61.8 ±6.4
FAqC2-3 (25)	149.2 ±16.1	56 ±12.4
**Controls**		
IMI (15)	153.3 ±15.8	66.6 ±12.3
VEH (100µl/10 g)	163 ±16.7	65.0 ±10.5

FAC1-1 and FAC1-2 (Organic fractions); FAqC2-1 and FAqC2-3 (Aqueous fractions); Imipramine (IMI 15.0 mg/kg), Tween 20 solution 1% (VEH). ANOVA, with post-test Dunnet with *p <0.05 (n = 6), in comparison with the VEH.

**Table 4:** Effect of different dose of active fractions of leaves from *Tilia americana *var. mexicana (Schltdl.) J. W. Hardin, on the parameters of total Crossings (TC) and rearings (R) of mice in the open field.

**Doses (mg/kg)**	**TC**	**R**	**TC**	**R**	**TC**	**R**	**TC**	**R**
	**FAC1-1**		**FAC1-2**		**FAqC2-1**		**FAqC2-3**	
6.25	115.8±11*	32.5 ±6*	103±16*	40.8±6*	136.3±20	48.5±5.4	113.6±14*	50.6±5.2
12.5	104.5±17*	38.3±10*	107±18*	43.3 ±6*	129.5±19	51 ±11	99.1±11.9*	50.3±9.6
25	97.3±13*	34.1±7*	101.8±5*	45±11*	129.7±9	61.7±6.4	149.2±15	51.4±9.6
50	99.8 ±13*	34.8±4*	106.8±23*	40±11*	130.6±20	44±10.8*	133±18.9	48.5±4.0
75	99.8±6*	45.8±9*	98.8±25*	44±10*	137.1±14	40.1±10.8*	121.8±16	51.1±13
**Controls**								
IMI (15)	153.3 ±15	66.6 ±12.3						
VEH 100µl/10g	163 ±16.7	65.0 ±10.5						

FAC1 (Organic fractions from AcOEt extract) y FAqC2 (Aqueous fractions from AcOEt extract); VEH= Tween 20 solution 1%; IMI=Imipramine 15 mg/kg. ANOVA with post-test Dunnet with *p <0.05 (n = 6), in comparison with the VEH.

**Table 5:** Effect of different doses of Tiliroside from *Tilia americana*, in mice on the open field (OFT).

**Til (mg/kg)**	**TC**	**R**
0.05	120.00 ±16.4*	40.00 ±8.10*
0.1	128.80 ±24.4*	44.20 ±8.05*
0.5	117.67 ±27.1*	38.40 ±10.1*
1.0	129.60 ±22.6*	48.17 ±5.02*
**Controls**		
IMI (15)	153.3 ±15	66.6 ±12.3
VEH (100µl/10 g)	163 ±16.7	65.0 ±10.5

Til= Tiliroside from *T. americana*; VEH = Tween 20 solution 1%; IMI=Imipramine 15 mg/kg. ANOVA with post-test Dunnet with *p <

0.05 (n = 6), in comparison with VEH; TC= Total Crossings (TC), R=rearings.

## Conclusion

The results of this study demonstrated that tiliroside and fractions with different chemical composition from *T. americana*, exert an antidepressant effect in the FST, TST, and sedative activity on OFT.
